# The Executive Control of Face Memory

**DOI:** 10.3233/BEN-2011-0339

**Published:** 2011-11-07

**Authors:** Steven Z. Rapcsak, Emily C. Edmonds

**Affiliations:** ^1^Department of NeurologyUniversity of ArizonaTucsonAZUSA; ^2^Neurology SectionSouthern Arizona VA Health Care SystemTucsonAZUSA; ^3^Department of PsychologyUniversity of ArizonaTucsonAZUSA

**Keywords:** Face memory, false recognition, frontal lobe damage, cognitive aging

## Abstract

Patients with frontal lobe damage and cognitively normal elderly individuals demonstrate increased susceptibility to false facial recognition. In this paper we review neuropsychological evidence consistent with the notion that the common functional impairment underlying face memory distortions in both subject populations is a context recollection/source monitoring deficit, coupled with excessive reliance on relatively preserved facial familiarity signals in recognition decisions. In particular, we suggest that due to the breakdown of strategic memory retrieval, monitoring, and decision operations, individuals with frontal lobe impairment caused by focal damage or age-related functional decline do not have a reliable mechanism for attributing the experience of familiarity to the correct context or source. Memory illusions are mostly apparent under conditions of uncertainty when the face cue does not directly elicit relevant identity-specific contextual information, leaving the source of familiarity unspecified or ambiguous. Based on these findings, we propose that remembering faces is a constructive process that requires dynamic interactions between temporal lobe memory systems that operate in an automatic or bottom-up fashion and frontal executive systems that provide strategic top-down control of recollection. Executive memory control functions implemented by prefrontal cortex play a critical role in suppressing false facial recognition and related source memory misattributions.

## 1. Introduction

The apparent ease with which we normally recognize familiar faces creates the impression of a largely automatic or bottom-up memory process that is relatively impervious to strategic top-down influences. Although we encounter hundreds of different individuals in our daily lives, a quick glance at the face is usually sufficient to determine its familiarity and to trigger the retrieval of associated identity-specific semantic or episodic information about the person. Thus, once set in motion by the face cue, the recognition process seems to unfold in a mandatory fashion without any obvious need or even opportunity for exercising deliberate cognitive control. Consistent with the presumption of automaticity that is considered the defining characteristic of modular systems [22], experimental studies have shown that familiar faces presented as task-irrelevant distractors cannot be intentionally ignored [32,38,55, 84] and also that face identity information continues to be processed in an obligatory manner even in the absence of conscious awareness [55,73,86,87].

Despite the impressive capacity, speed, and efficiency of face memory, the recognition process is by no means infallible. For instance, Young, Hay, and Ellis [85] documented a surprising variety of errors in cognitively normal individuals who were asked to keep a diary of the difficulties they experienced in recognizing people in everyday life. These records included both errors of omission, when a familiar person was not recognized, and errors of commission when an unfamiliar individual was mistaken for someone familiar. There were also many instances of partial recognition when the face seemed familiar but the diarists were unable to recall additional identity-specific details about the person thus failing to establish the appropriate context for the sense of familiarity. The inaccuracy of face recognition memory is also dramatically illustrated by the notorious unreliability of eyewitness testimony where errors can have serious consequences, including the failure to convict the guilty or the wrongful conviction of an innocent person [2,6]. Recognition errors in healthy

ISSN 0953-4180/11/$27.50 © 2011 – IOS Press and the authors. All rights reserved individuals have important theoretical implications because they demonstrate that bottom-up reactivation of stored identity-specific knowledge by the face cue does not always occur in an all-or-none fashion and that the information automatically retrieved from memory may be incorrect, irrelevant, ambiguous, or underspecified. In addition, the fact that the participants in the Young et al. [85] diary study were aware of their recognition errors and could also frequently correct them suggests that normal subjects routinely monitor the output of the face recognition system and can engage strategic top-down memory retrieval and decision operations whenever there is uncertainty about the real identity of the person encountered.

Neurological damage can greatly exaggerate the types of difficulties experienced by cognitively intact individuals in recognizing faces. From a clinical perspective, face recognition impairments in neurological patients can manifest either as memory loss or memory distortion [59,60,66]. The behavioral hallmark of the former is the striking failure to identify *familiar* faces, whereas the latter is characterized by false recognition or misidentification of *unfamiliar* faces. Although in some individuals both omission and commission errors are observed, current evidence suggests that these qualitatively distinct face recognition disorders are dissociable and have different cognitive mechanisms and neural substrates. In particular, the inability to recognize familiar faces is associated with damage to temporal lobe memory systems that are responsible for encoding long-term visual memory representations for faces and for linking these records with other verbal and nonverbal person-specific information (e.g., voice, name, pertinent biographic/contextual details) represented in different neocortical regions into a unified memory trace. The critical neural substrates of this cross-modal associative memory process that normally allows the face of a familiar individual to reactivate stored person-specific knowledge in an automatic or bottom-up fashion via pattern completion [54] mechanisms include the fusiform face area (FFA), anterior temporal cortex (ATC), and medial temporal lobe regions (hippocampus and related structures) [59,60]. Damage to the FFA has been implicated in prosopagnosia, a modality-specific visual memory disorder in which familiar individuals cannot be recognized from their faces but patients retain the ability to retrieve identity-specific information from non-visual cues (e.g., when hearing the person's voice or name) [13,75]. By contrast, damage to anterior and medial temporal lobe regions results in multimodal deficits of person recognition in which stored knowledge about familiar people cannot be accessed regardless of the sensory modality or verbal/nonverbal nature of the memory cue (i.e., face, voice, or name) [23].

While memory loss for familiar faces is associated with temporal lobe lesions, false recognition and misidentification of unfamiliar faces is observed in patients with damage to prefrontal cortex [61–66]. In order to explore the cognitive mechanisms responsible for these memory distortions, in this paper we review pertinent neuropsychological findings from two subject populations with increased susceptibility to false facial recognition: patients with focal frontal lobe lesions and normal elderly individuals experiencing an age-related decline in frontal executive function. Collectively, these data provide converging evidence that face memory illusions reflect the breakdown of strategic memory retrieval, monitoring, and decision functions implemented by prefrontal cortex that play a critical role in attributing the experience of facial familiarity to a specific context or source. Based on these empirical observations, we propose a neuropsychological model of face recognition which postulates that remembering faces is a dynamic and constructive process that requires reciprocal functional interactions between temporal lobe memory systems that operate in a mandatory or bottom-up fashion and frontal executive systems that are involved in the strategic top-down control of recollection.

## 2. False recognition and misdentification of faces in patients with frontal lobe lesions

False facial recognition/misidentification can be defined as a type of memory distortion in which patients mistakenly believe that novel faces had been encountered previously (i.e., that they are familiar, famous, or personally known). Neuropsychological studies have shown that such misattributions of facial familiarity can take different clinical forms. In many cases, memory illusions are revealed by the strong tendency to produce false alarms to novel distractor faces in anterograde memory experiments. For instance, Rapcsak et al. [64, 66] demonstrated that patients with focal right frontal lobe lesions produced substantially more false alarms in yes/no face recognition memory tests than patients with equivalent or worse face memory impairment due to right temporal lobe damage (Fig. 1). These findings provided evidence that face memory distortions in frontal patients are not simply the result of memory loss and suggested that the increased propensity

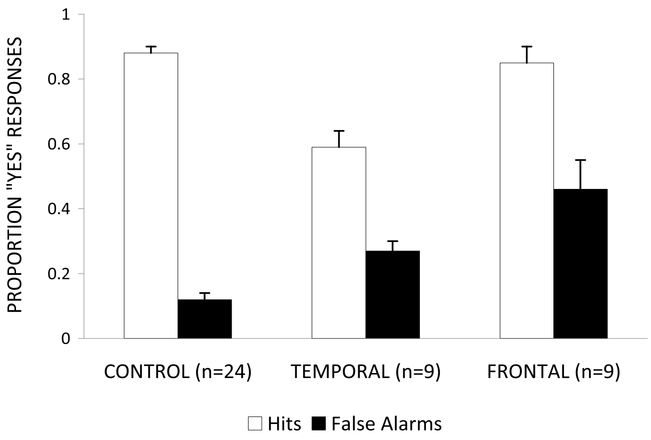

Fig. 1. Anterograde face memory performance demonstrating elevated false alarm rates in patients with right frontal lobe lesions compared to normal controls and patients with right temporal lobe damage (modified from Rapcsak, Nielsen, Glisky, and Kaszniak, 2002).

for false recognition may instead be related to the use of inappropriate decision strategies under conditions of reduced memory discrimination. Specifically, it has been proposed that both familiarity and recollection can contribute to recognition memory decisions [29,42,83]. Whereas judgments based on stimulus familiarity are relatively automatic, recollection frequently requires an effortful and strategic memory search to retrieve specific contextual information pertaining to the presentation of the target item during the study episode. Context recollection is likely to play a particularly important role in recognition memory tests in which targets and distractors are highly similar and therefore difficult to distinguish on the basis of familiarity alone [52]. When participants are unable to access distinctive contextual information about individual test items, however, they may respond on the basis of general of "gist" memory for the study episode [6]. According to "fuzzy trace theory," gist memory representations are automatically extracted during the study phase, in parallel with item-specific memory traces, and preserve categorical information about what study items had in common [6]. The critical point to note is that the general similarity information encoded in gist memory traces cannot reliably discriminate between targets and category-consistent distractors at test because both types of recognition probes match this generic memory description of the study episode. Consequently, the sense of familiarity associated with the activation of gist or category-level memory representations by novel distractor items is considered the principal source of false alarms in recognition memory experiments [6]. It has also been demonstrated, however, that by relying consistently on the recollection of item-specific contextual information to support a positive recognition decision normal individuals can oppose and inhibit the influence of gist or category-based familiarity and thereby suppress false recognition [6,17,69]. Thus, whereas the automatic activation of gist or categorical memory representations by novel distractors promotes false recognition, strategic recollection of contextual details about individual test items serves to reduce memory distortions.

Based on these theoretical considerations, Rapcsak et al. [64,66] proposed that false recognition errors in frontal patients in anterograde face memory tests resulted from over-reliance on category-level memory representations that cannot support the recognition of individual identity. In particular, they suggested that patients with frontal lobe damage may have had difficulty encoding distinct, non-overlapping memory representations for the individual faces presented but were able to automatically extract and store categorical information about the common attributes of study items (e.g., that the faces were yearbook photographs of young students). Without access to detailed contextual information linking test items to the study episode, patients could not reliably discriminate between targets and category-consistent distractors (i.e., the faces of other young students) because both types of recognition probes matched the category-level memory representation and thus gave rise to feelings of familiarity. It is possible however, that in addition to (or instead of) defective encoding frontal patients were impaired in their ability to initiate and conduct a strategic memory search to retrieve specific contextual information about test items. Furthermore, the striking failure of these patients to realize that the absence of specific recollection is inconsistent with a positive recognition decision suggested a breakdown of executive memory monitoring and criterion-setting functions. Specifically, it appeared that instead of carefully evaluating memory traces evoked by test items for contextual details pertaining to the study episode, frontal patients adopted lenient decision criteria and accepted familiarity signals based on general category resemblance as conclusive evidence of prior encounter. Presumably, normal individuals are able to oppose and suppress false recognition errors to category-consistent distractor faces by insisting that recollection of item-specific contextual information accompany a positive recognition decision [64,66]. The failure to use recollection to inhibit the influence of automatically activated category-level memory representations on recognition judgments, therefore, is likely to have played an important role in the pathogenesis of face memory distortions in patients with frontal lobe damage.

The hypothesis that false recognition errors in frontal patients reflected an over-reliance on categorical memory representations received empirical support from the results of face memory paradigms that included both category-consistent and category-inconsistent distractors. In particular, Rapcsak et al. [64,66] showed that following exposure to pictures of white male faces in the study phase of a memory experiment frontal patients were much more likely to false alarm to category-consistent distractors (i.e., other white males) than to category-inconsistent distractors that differed from target faces in terms of race or gender (i.e., non-white males, or white females). The finding that the majority of false alarms occurred to category-consistent distractors provided confirmation that frontal patients had preserved access to categorical information about study items, while retaining impoverished memory traces for the individual faces presented, and that they used this general memory description of the study episode in their recognition decisions. In fact, the performance of frontal patients in these memory experiments demonstrated all the key behavioral signatures of categorical processing: preserved between-category but poor within-category discrimination, and a strong propensity for similarity-based generalization to novel items [48, 49,67].

Face memory distortions in patients with frontal lobe lesions are not necessarily limited to excessive false alarms in anterograde memory experiments. In particular, several patients have been described who showed increased false recognition in retrograde memory paradigms that involved judging whether they had ever seen a novel face before, or required familiarity/fame decisions using sets of stimuli that included both famous and unfamiliar faces [61–66]. In these experimental situations, the spurious sense of familiarity triggered by novel faces was occasionally accompanied by the retrieval of false biographic information resulting in frank misidentifications. Note that the cognitive requirements of retrograde face memory paradigms are similar to what people must do to reach recognition decisions in everyday life: assess the familiarity of the face and attempt to retrieve unique, identity-specific contextual information about the person encountered. In fact, patients with frontal lobe damage sometimes demonstrate false recognition/misidentification in real-life settings by mistaking unfamiliar individuals for famous people or personal acquaintances [61–63,81,82].

Neuropsychological evidence suggests that false recognition in the anterograde and retrograde domains of face memory involve similar underlying cognitive mechanisms. Thus, consistent with results obtained in anterograde memory experiments, Rapcsak et al. [64, 66] found that patients with right frontal lobe damage produced more false alarms than normal controls and patients with right temporal lobe lesions in a retrograde familiarity decision test that included famous faces and novel faces with a celebrity-type appearance as stimuli (Fig. 2). By contrast, hit rates and correct identifications to famous faces were reduced in patients with temporal lobe lesions compared to patients with frontal lobe damage who did not significantly differ from controls (Fig. 2). Once again, these findings underscore the important theoretical point that false facial recognition in frontal patients cannot be fully explained by face memory loss. Instead, Rapcsak et al. [64,66] proposed that the inappropriate reliance on readily accessible category-level memory representations that contributed to false alarms in anterograde memory experiments was also responsible for false recognition errors in retrograde face memory tasks. For example, patients with frontal lobe damage may incorrectly claim that a novel face is familiar/famous based on strong categorical resemblance to facial prototypes that we associate with celebrity status (e.g., "actress-type" face). These prototypes represent our general knowledge of faces and are the automatic product of a gist-based memory process that extracts and preserves information about shared facial attributes of individuals belonging to different social groups. Novel faces that closely resemble stored prototypes may evoke a strong feeling of familiarity and thus give rise to memory illusions. Consistent with the hypothesis that false recognition is based on the automatic activation of categorical face memo-

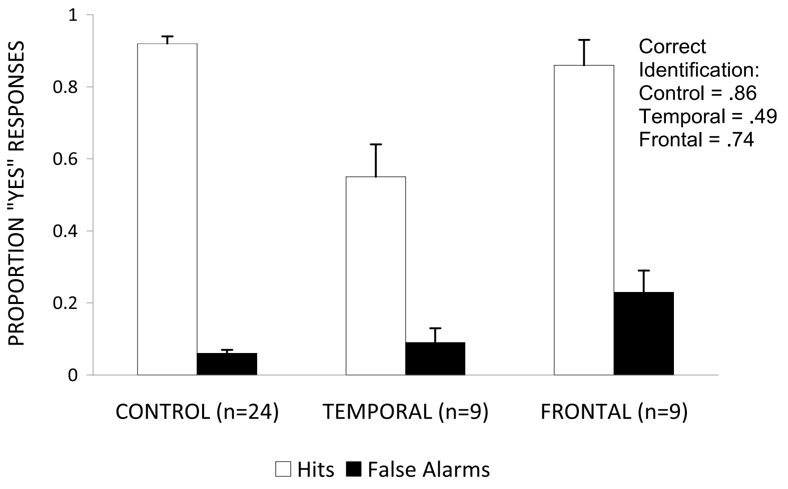

Fig. 2. Performance on a retrograde face familiarity decision test that included famous faces and novel faces with a celebrity-type appearance as stimuli. False alarms to unfamiliar faces are increased in patients with right frontal lobe lesions, whereas hit rates and correct identifications to famous faces are reduced in patients with right temporal lobe damage (modified from Rapcsak, Nielsen, Glisky and Kaszniak, 2002).

ry representations and the retrieval of associated general knowledge about social categories or stereotypes, frontal patients typically use generic descriptions to justify their claim that a novel person is familiar/famous (e.g., "saw her on TV"). However, the general sense of familiarity associated with the activation of category prototypes by novel faces can normally be opposed by engaging in a strategic memory search to retrieve additional biographic information about the person (e.g., by attempting to recollect specific movies the "actress" appeared in or by trying to remember her name). Thus, cognitively intact individuals can suppress false recognition by relying on the presence or absence of detailed identity-specific contextual information as the appropriate criterion for making face memory decisions [64]. By contrast, frontal patients do not seem to engage in effortful and strategic context retrieval and are likely to respond on the basis of general facial familiarity that is the undifferentiated output and automatic product of category-level face processing. Furthermore, due to defective monitoring, patients may fail to realize that the absence of person-specific contextual information is inconsistent with a positive recognition decision and consequently mistake "looking famous" for "being famous." Empirical support for the hypothesis that face memory distortions reflect the use of inappropriate decision criteria was provided by demonstrations that false recognition errors in frontal patients can be dramatically reduced by asking them to call faces familiar/famous only if they could explicitly remember the profession and name of the person [64]. These observations suggest that, although they may fail to do so spontaneously, some frontal patients retain the ability to use the output of the context retrieval process to suppress false recognition errors once the appropriate memory decision strategy is revealed to them.

To summarize, patients with frontal lobe damage demonstrate increased susceptibility to memory illusions in both anterograde and retrograde tests of face recognition memory. However, in neither memory domain can false recognition be entirely accounted for by face memory loss. Instead, the neuropsychological evidence suggests that face memory distortions reflect the breakdown of strategic memory retrieval, monitoring, and decision functions critical for attributing facial familiarity to a specific context or source. Due to the failure to encode, retrieve, or make appropriate use of identity-specific contextual information, patients with frontal lobe damage are unable to oppose and inhibit recognition decisions based on general familiarity signals that result from the automatic activation of preserved category-level face memory representations. In addition, on those occasions when patients do attempt to engage in strategic memory retrieval, the faulty search process may assign facial familiarity to an incorrect or inappropriate source resulting in misidentifications. The patients' willingness to uncritically accept false and often implausible identity-specific contextual information is consistent with the proposed impairment of executive memory monitoring and verification functions. Additional evidence of defective monitoring comes from observations that frontal patients with false recognition/misidentification exhibit anosognosia for their face memory impairment and have difficulty judging the accuracy of their recognition decisions [56, 61–63].

## 3. Face memory distortions in cognitively intact older adults

The literature on cognitive aging suggests that face memory illusions may be particularly common in the elderly. Specifically, a number of studies have reported that older adults show elevated false alarm rates to novel faces in yes/no face recognition memory tests compared to younger adults [1,3,4,37,47,74]. Similar to the neuropsychological explanations offered to account for memory distortions in frontal patients, false facial recognition in older individuals has been attributed to an age-related deficit in context recollection and a corresponding increase in the use of familiarity when making face memory decisions [1,3, 47,64,74]. Furthermore, it has been suggested that the context-recollection deficit responsible for the increased susceptibility of older adults to face memory illusions reflected an age-related decline in frontal lobe function [5,18,64]. This theoretical formulation is consistent with other evidence that automatic memory processes such as familiarity remain relatively invariant with age while more controlled memory processes such as recollection and source monitoring show age-related decline [28,30,33,76,83], and also with neuroimaging studies demonstrating prominent changes in frontal lobe structural/functional integrity with increasing age [8,15,27,78].

To test the context-recollection deficit hypothesis of the age-related increase in face memory distortions, we [18] recently conducted a study comparing the performance of 40 old (mean age = 74.0, range = 63–86) and 40 young (mean age = 20.8, range = 18–28) cognitively intact adults on a face memory paradigm originally developed by Bartlett et al. [5]. This recognition memory task included three different types of distractor items with varying levels of familiarity: "familiarized lures" that were exact repetitions of faces that participants were exposed to in a personality rating task conducted prior to the presentation of the study list, "conjunction lures" that were synthetic faces created by recombining the inner regions (eyes, nose, and mouth) and the outer regions (hair, ears, and jaw line) from two different study faces, and entirely new faces. It was assumed that perceived levels of familiarity would be relatively low for novel distractor faces, intermediate for conjunction faces that shared features with study items, and highest for familiarized lures that were exact repetitions of items encountered earlier in the experiment but in a different context than targets. Therefore, if memory distortions were primarily triggered by familiarity signals, then false recognition errors would be expected to track the gradient of facial familiarity that existed across the different distractor types (familiarized lures > conjunctions > novel faces). Test instructions specified that in making recognition judgments participants should say "yes" only to faces that appeared on the study list and "no" to all other items, and subjects were explicitly warned that memory probes will include not only study stimuli and novel faces but also conjunctions and familiarized lures. Because both targets and familiarized lures were exact repetitions of faces presented earlier the experiment they had equivalent levels of familiarity and could only be discriminated by recollecting the specific context in which the items were originally encountered (i.e., by remembering whether the face appeared on the study list or whether it was presented during the personality rating task). By contrast, conjunction lures and novel faces could be distinguished from each other, as well as from both targets and familiarized lures, solely on the basis of differences in the strength of the familiarity signal. Applied to this face memory paradigm, the context-recollection deficit hypothesis of the age-related increase in false recognition errors makes relatively straightforward predictions. Specifically, we should find substantial age differences in false recognition rates to familiarized lures, as older adults are expected to be less successful than younger adults in using context recollection to oppose and suppress false alarms to these highly familiar stimuli. Furthermore, in the absence of recollection, false alarms to familiarized lures in older adults should approach hit rates to study faces because these two types of items are equivalent in terms of their levels of familiarity. By contrast, the context-recollection deficit hypothesis predicts minimal age differences in false recognition to conjunction lures and entirely new faces, since memory decisions to these items can rely on the automatic assessment of stimulus familiarity that should be relatively preserved in older adults.

As can be seen from Fig. 3, the results of this experiment provided strong support for the context-recollection deficit hypothesis of false facial recognition. In particular, a significant age effect was found with older adults demonstrating markedly increased false alarm rates in comparison to younger adults for familiarized lures that required accurate recollection of contextual information to reject. False alarms to fa-

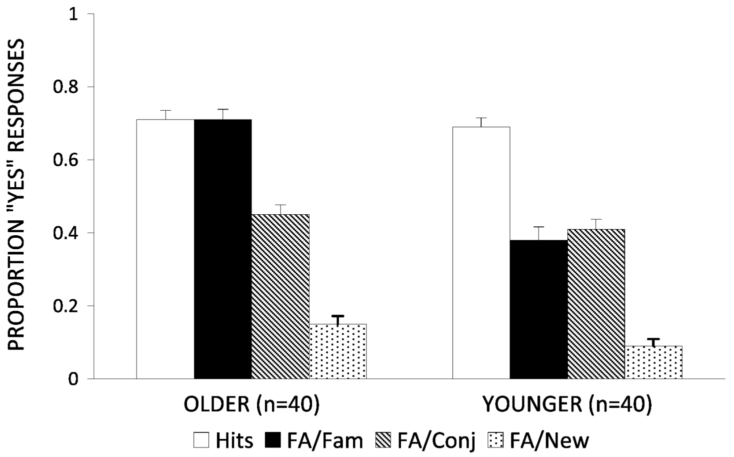

Fig. 3. Hit and false alarm rates on an anterograde face memory test in older and younger adults (modified from Edmonds, Glisky, Bartlett, and Rapcsak, under review). FA/Fam = false alarms to familiarized lures, FA/Conj = false alarms to conjunction faces, FA/New = false alarms to novel faces. A significant age-related increase is observed for FA/Fam.

miliarized lures in older adults were indistinguishable from hit rates to study faces, suggesting that participants could not reliably use context recollection to discriminate between these two types of memory probes that were equivalent in terms of their levels of familiarity. By contrast, there were no significant age differences in false alarms to conjunction lures that recombined parts of study list faces, or to entirely new faces. Overall, the pattern of false recognition errors observed in older adults was consistent with excessive reliance on a familiarity-based response strategy. Specifically, in the absence of context recollection older adults seemed to base their memory decisions on the strength of the facial familiarity signal, as evidenced by a linear increase in false alarm rates with increasing familiarity of the lures.

To summarize, a number of studies have reported that older adults show increased susceptibility for face memory illusions, possibly reflecting an age-related decline in frontal executive memory functions. While direct confirmation of this hypothesis will require correlating false facial recognition with neuroimaging measures of age-related structural/functional changes in prefrontal cortex, there are some obvious behavioral similarities to the face memory distortions documented in patients with focal frontal lobe lesions. In particular, false recognition errors in both subject populations seem to reflect a deficit in context recollection/source monitoring, combined with excessive reliance on a familiarity-based strategy in face memory decisions. These findings provide converging evidence that strategic top-down context retrieval and monitoring functions are critical for opposing and inhibiting the potentially misleading bottom-up influence of facial familiarity on recognition memory decisions and reinforce the notion that these frontal executive control operations constitute the principal cognitive mechanism for suppressing false recognition and related source memory misattributions. From an applied perspective, the increased vulnerability of cognitively normal elderly individuals to face memory illusions has important implication for eyewitness testimony. Specifically, the results of our study and similar findings in the literature [2,47,74] suggest that older adults are likely to experience difficulty in attributing the experience of facial familiarity to the correct source. As a result, elderly persons may have trouble determining whether an individual is familiar because he/she was encountered at the scene of a crime or in another neutral context (i.e., source memory confusions). Furthermore, elderly individuals may show increased susceptibility for falsely recognizing innocent suspects based on categorical facial attributes they share with the true perpetrator of the crime (e.g., gender, age, race). The marked propensity for face memory distortions could also have negative implications for the social or interpersonal functioning of older adults in everyday life.

## 4. Discussion

To provide a theoretical framework for interpreting face memory distortions associated with frontal lobe dysfunction, in Fig. 4 we present a modified version of the cognitive model of face recognition originally proposed by Bruce and Young [7]. According to the

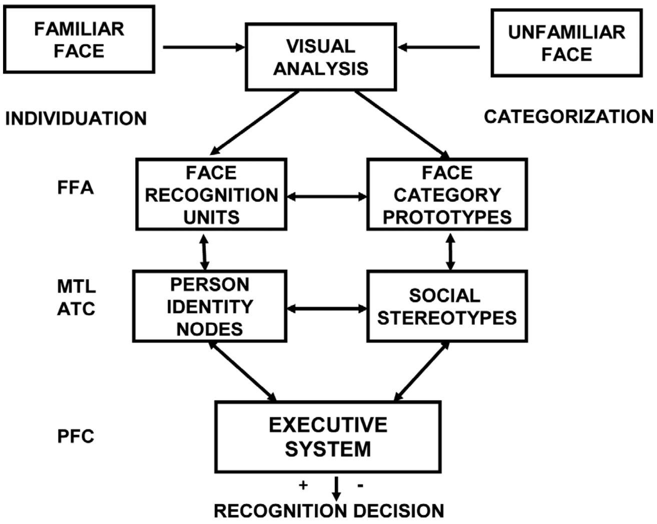

Fig. 4. Neuropsychological model of face recognition memory. The automatic bottom-up output of temporal lobe memory systems, representing the combined product of individuation and categorization, is monitored, verified, and integrated by the frontal executive system that exerts strategic top-down control over the recognition process. FFA = fusiform face area, MTL = medial temporal lobe, ATC = anterior temporal cortex, PFC = prefrontal cortex.

revised model, face recognition memory performance reflects the dynamic interaction of two closely related cognitive operations that involve processing faces at different levels of specificity: individuation and categorization [21,41,43,59]. Individuation makes use of facial information to recognize *familiar* people at the most specific taxonomic level corresponding to unique personal identity. A critical early component of the individuation process is the activation of "face recognition units" (FRUs) that store distinct visual memory representations for familiar faces (Fig. 4). Activated FRUs give rise to a graded sense of familiarity, the strength of which is determined by the degree of perceptual overlap between the face cue and the stored memory trace. The engagement of FRUs is normally followed by the retrieval of identity-specific semantic or episodic information from memory via activation of "person identity nodes" (PINs) that provides the appropriate context for the feeling of familiarity evoked by the face. Note that the sequential activation of FRUs and PINs corresponds to two distinct memory states: the initial detection of facial familiarity followed by the retrieval of associated contextual information that specifies the source of familiarity. This conceptualization is consistent with the central tenet of dual-process models that postulate two different forms of memory contributing to recognition judgments: a fast and automatic familiarity process and a relatively slow and controlled process that involves context recollection [83]. In fact, the difference between these memory operations can be conveniently illustrated by the common experience of immediately recognizing that a face is familiar without being able to recollect who the person is or retrieve other pertinent contextual details regarding prior encounters [42,83].

The functional integrity of the individuation process is essential for the rapid and accurate identification of familiar people that characterizes normal face recognition performance. By relying on distinct, nonoverlapping memory representations, individuation allows for maximum differentiation or pattern separation [45,52–54] between memory traces and thus avoids the problem of interference that could lead to mistaking one familiar person for another. It is important to emphasize that the efficiency and ultimate success of the individuation process is strongly influenced by the frequency of prior exposure to a particular person. Whereas identity-specific contextual information about highly familiar individuals is automatically reactivated in a bottom-up fashion by the face cue via pattern completion mechanisms, recognizing people we are less familiar with can create difficulties as our stored knowledge of these persons is not as well consolidated and the associative links between facial and other types of person-specific information are not firmly established. Identifying individuals we encounter only infrequently may require the engagement of effortful and strategic top-down memory retrieval operations because the mnemonic information automatically triggered by the face cue is likely to be incomplete or ambiguous with respect to context or source. Furthermore, because memory traces for relatively unfamiliar people are not well differentiated and are likely to contain fewer distinctive person-specific attributes, they are also more susceptible to interference from representations of other similar individuals in memory.

Individuation can be contrasted with recognition memory processes that are primarily used to classify *unfamiliar* people into meaningful social categories based on their facial appearance [21,41,43,59]. Categorization takes advantage of pre-existing knowledge about facial characteristics shared by members of distinct social groups that are stored in memory as abstract perceptual prototypes (Fig. 4). In contrast to individuation that relies on pattern separation to maximize differences between memory traces, face category prototypes are the result of a gist-based memory process that relies on pattern integration mechanisms [54] to extract and preserve general similarity information about structural attributes common to many faces [11,40,79]. Novel faces can be assigned to one or more relevant categories based on their degree of resemblance to stored prototypes. Category assignment typically reflects some combination of gender, age, and race, but it can also involve more complex social inferences based on facial attributes we associate with certain occupations (e.g., actress, politician) or personality traits (e.g., honesty, intelligence) [36]. The activation of face category prototypes provides access to general semantic knowledge corresponding to social stereotypes that contain information about attributes presumably shared by members of various groups (Fig. 4). Although category-based recognition mechanisms offer the benefit of simplifying social interaction by allowing pre-existing knowledge to generalize to novel individuals, the procedure has significant limitations due to its lack of differentiation. Specifically, because of the emphasis on shared structure and reliance on overlapping distributed memory representations [54,67], category-level recognition processes manifest a strong propensity for similarity-based generalization and therefore cannot support the recognition of individual identity. Furthermore, as we have seen, false recognition of novel individuals may occur when familiarity signals associated with the activation of category-level memory representations are misattributed as evidence of specific prior exposure to the person [64,66].

According to our model, individuation and categorization are complementary recognition processes implemented within temporal lobe face memory systems capable of operating at different levels of specificity (Fig. 4). Although distinct in terms of computational requirements, under normal circumstances the two recognition procedures that represent the specific and general components of face memory are assumed to be highly interactive. Specifically, when attending to the face of a person both processes are automatically initiated in a bottom-up fashion and both types of information are available as outputs for flexible use in recognition memory judgments. Thus, we propose that temporal lobe memory systems obligatorily encode and retrieve both the individuating information that supports true recognition and the categorical similarity information that is the primary source of false recognition errors to novel faces. As shown in Fig. 4, the critical components of the temporal lobe face recognition memory network include the FFA, ATC, and MTL. The FFA is likely to carry out the neural computations assigned to FRUs, whereas ATC/MTL are responsible for cross-modal binding and integration of facial and other types of verbal/nonverbal person-specific knowledge and thus may represent the neural substrates of PINs (Fig. 4). Furthermore, current evidence suggests that hippocampus vs. temporal lobe neocortex may make distinct contributions to recollection vs. familiarity-based recognition and thus may also differentially contribute to individuation versus categorization. In particular, the hippocampus is thought to play a critical role in recollection by binding item and context/source information using pattern separation algorithms that maximize differences between memory traces thereby reducing interference and mnemonic competition [20, 44,45,52–54,83]. Therefore, the hippocampus may be an important neural substrate of the individuation process that leads to the establishment of unique, non-overlapping associative memory representations for familiar persons. By contrast, perirhinal cortex and other temporal lobe neocortical regions have been implicated in familiarity-based recognition processes [20,44, 45,52–54,83]. The neural computations performed by temporal lobe neocortex may include pattern integration mechanisms to encode general similarities across memory traces [44,45,52–54,67]. The overlapping distributed memory representations established in neocortex may thus support the categorization process that enables generalization of pre-existing knowledge to novel individuals.

The neuropsychological model shown in Fig. 4 also postulates the existence of a frontal executive component that interacts with and exerts strategic top-down control over the operations of temporal lobe face memory systems [59,60,64,66]. Control procedures implemented by prefrontal cortex (PFC) include the monitoring, verification, and integration of the bottom-up information automatically retrieved from memory in response to the face cue, representing the combined output of the individuation and categorization process, and the setting of appropriate response criteria for the recognition decision. Effective monitoring requires the critical evaluation of the evidence provided by activated memory traces, taking into consideration both the strength of the familiarity signal and the amount and quality of the identity-specific contextual information recovered. It is assumed further that when the obligatory cue-dependent output of temporal lobe memory systems is deemed insufficient for reaching a definite conclusion about a person's identity, PFC can initiate a strategic memory search to retrieve additional information required to establish the appropriate context for the sense of familiarity. In short, we propose that PFC is involved in implementing strategic top-down context retrieval, monitoring, and decision functions to ensure that the experience of facial familiarity is attributed to the correct source. Extensive monitoring and memory control operations are not usually required for the recognition of highly familiar faces because for these individuals distinctive contextual information is automatically reactivated in a cue-dependent or bottom-up fashion. However, the strategic engagement of executive resources becomes essential under conditions of uncertainty when the face cue does not directly elicit relevant identity-specific contextual information, leaving the source of familiarity unspecified or ambiguous (e.g., when we are not sure whether a familiar-looking face is somebody we know) [64].

As we have seen, frontal lobe dysfunction does not produce the type of severe face memory loss observed in prosopagnosia or in patients with multimodal person recognition impairment following damage to temporal lobe memory systems. Instead, frontal lobe damage or age-related functional decline is associated with increased vulnerability to memory distortions that include false recognition/misidentification of unfamiliar faces. The neuropsychological studies reviewed in this paper converge on the conclusion that the core functional impairment underlying face memory illusions is a context-recollection/source monitoring deficit, coupled with inappropriate reliance on relatively preserved facial familiarity signals in recognition memory judgments. In particular, the evidence presented here suggests that due to the breakdown of strategic top-down context retrieval and monitoring functions, individuals with frontal lobe dysfunction did not have a reliable mechanism for opposing and inhibiting the potentially misleading bottom-up influence of facial familiarity on recognition decisions, leaving them susceptible to source memory confusions. Specifically, we have shown that participants with frontal lobe impairment had difficulty determining whether their subjective experience of remembering was based on memory for specific faces (i.e., reflecting the output of the individuation process) or memory for faces in general (i.e., reflecting the output of the categorization process), and they performed poorly on recognition tasks in which correct memory decisions to previously encountered faces required accurate recollection of the specific experimental context in which the individual items were originally presented. Furthermore, when diagnostic source memory information was not immediately available, frontal patients seemed unable to generate appropriate retrieval strategies with which to interrogate and explore the contents of temporal lobe memory systems. As a result, they could not initiate and maintain an organized memory search to recover missing identity-specific contextual details and, due to the proposed monitoring deficit, could not verify whether the information retrieved from memory by the faulty search process was veridical or false. Taken together, these observations suggest that the primary role of PFC in face memory is to promote and supervise the strategic encoding, retrieval, and monitoring of individuating contextual information essential for accurate source memory attributions.

Consistent with the neuropsychological data presented here, imaging studies in normal subjects have demonstrated PFC activation in both anterograde and retrograde face memory paradigms [35,39,46], as well as during working memory tasks that required the active maintenance and manipulation of face memory information [16,58]. Collectively, these results support the view that remembering faces depends on dynamic functional interactions between temporal lobe memory and frontal executive systems (Fig. 4). Furthermore, imaging evidence that levels of neural activity within temporal lobe face processing areas are modulated by top-down signals originating in PFC [16,24,34,58] is consistent with the notion that the frontal lobes exert executive control over the recognition process (Fig. 4). Input from PFC may serve to bias processing in temporal lobe face memory networks toward the retrieval of task-relevant information while at the same time suppressing the distracting influence of task-irrelevant or competing memory representations. For instance, when the face memory task calls for the recognition of individual identity, automatically activated categorical information is irrelevant and needs to be inhibited. In these situations, top-down signals from PFC may selectively enhance the retrieval of individuating face memory information while at the same time limiting interference by suppressing mnemonic competition from category-level memory representations. According to this view, executive functions implemented by PFC improve recognition accuracy by increasing the signal-to-noise ratio within temporal lobe face memory systems [66]. Strategic top-down modulation of the relationship between individuation and categorization by PFC is likely to play an important role in maintaining the dynamic flexibility of the face recognition system required for operating efficiently in everyday social situations that typically involve multiple encounters with both familiar and unfamiliar people.

Our neuropsychological account of false facial recognition has several features in common with other theoretical models that have implicated defective strategic context recollection and source-monitoring failure in the pathogenesis of memory distortions [25,26,31, 33,50,51,71,72]. The findings discussed here are also consistent with other reports of increased false recognition in patients with frontal lobe lesions [9,10,12, 14,57,68,80] and in normal elderly individuals [30, 70] demonstrated on both verbal and nonverbal memory tasks. Along these lines, it is important to emphasize that although we focused here on the role of PFC in face memory, we do not wish to suggest that false recognition in individuals with frontal lobe dysfunction is necessarily domain-specific. In our experience, the tendency of frontal patients to produce false alarms is determined more by task demands than by stimulus category and is likely to be observed in any anterograde memory test that requires item-specific recollection to discriminate between targets and similar category-consistent distractors [19]. The general propensity for false recognition under these test conditions points to a common functional impairment contributing to memory distortions across a wide range of experimental stimuli: defective item-specific recollection with inappropriate reliance on preserved gist or category-level memory representations. Similarly, it has been demonstrated that some frontal patients produce false recognition/misidentification errors not only to faces but also to unfamiliar names or voices in retrograde memory paradigms involving familiarity/fame judgments [61,62,81,82]. Collectively, these observations suggest that, consistent with the functional characteristics of central systems [22], PFC plays a domain-general role in the executive control of memory. Elucidating how the various memory control operations discussed here (e.g., strategic encoding/retrieval, source monitoring, verification, inhibition of interference by task-irrelevant or competing memory representations) map onto distinct subdivisions within PFC remains an important challenge for future investigations and will require converging evidence from lesion-deficit correlations in neurological patients and functional imaging studies in normal individuals. Unfortunately, our data cannot be used to address the contribution of different frontal regions to the executive control of face memory because the lesions in our patients were typically large and extended across multiple cortical subdivisions. However, with respect to the issue of laterality, our results indicate that although both left and right frontal lobe lesions can produce face memory impairment, excessive false recognition is primarily observed in patients with right PFC damage [65]. Additional insight into the operations of the distributed face recognition network could be provided by imaging studies directly comparing patterns of activation and changes in functional connectivity between distinct temporal lobe memory and frontal executive regions during recollection vs. familiarity-based face memory decisions, individuation vs. categorization [43,77], and true vs. false facial recognition. Finally, we note that the neuropsychological model postulating separate temporal lobe and frontal lobe contributions to face memory (Fig. 4) has received indirect support from recent studies of face recognition in normal subjects that used principal component analyses to identify two independent components: one serving to increase hits (temporal lobe memory) and the other to reduce false alarms (frontal executive) [5].

## Acknowledgments

This work was supported by NIH grant P30AG 19610. We thank CG for her assistance with the manuscript.
